# Evaluation of the Diagnostic and Prognostic Potential, and Correlation with the Tumor Immune Microenvironment of a Six-miRNA Signature Associated with Platinum Sensitivity in Patients with Early-Stage Non-Small-Cell Lung Cancer

**DOI:** 10.3390/cancers18203351

**Published:** 2026-10-16

**Authors:** Maria Mortoglou, Chara Papadaki, Aristeidis E. Boukouris, Kleita Michaelidou, Eleftherios Vorrias, Maria Kamaratou, Georgios Koronakis, Eleni Lagoudaki, Anastasios Koutsopoulos, Dimitrios Mavroudis, Sofia Agelaki

**Affiliations:** 1 Laboratory of Translational Oncology, School of Medicine, University of Crete, 71500 Heraklion, Greece; mmortoglou@med.uoc.gr (M.M.); chapapadak@uoc.gr (C.P.); arisbouk@gmail.com (A.E.B.); k.michailidou@uoc.gr (K.M.); eleftherios.vorrias@goc.com.cy (E.V.); medp2012275@med.uoc.gr (M.K.); koronakisgeorge@gmail.com (G.K.); mavroudis@uoc.gr (D.M.); 2 Department of Medical Oncology, University General Hospital of Heraklion, 71500 Heraklion, Greece; 3 Department of Pathology, University General Hospital of Heraklion, 71500 Heraklion, Greece; elagoudakimd@med.uoc.gr (E.L.); akoutsop@med.uoc.gr (A.K.); 4 School of Medicine, University of Crete, 70013 Heraklion, Greece

**Keywords:** NSCLC, microRNAs, chemotherapy, therapeutic resistance, platinum-based chemotherapy, non-coding RNAs

## Abstract

Background/Objectives: Platinum-based chemotherapy remains a standard-of-care treatment for non-small-cell lung cancer (NSCLC), yet its efficacy is influenced by multiple factors, such as the functionality of DNA damage response pathways. DNA-damaging agents, like cisplatin, contribute to the generation of an “inflamed” tumor microenvironment (TME), which in turn correlates with an active antitumor immune response. MicroRNAs (miRNAs), a family of small, non-coding RNAs, have emerged as critical post-transcriptional regulators of gene expression, playing vital roles in modulating DNA Damage Repair (DDR) pathways and sensitivity to cisplatin. Their functional involvement in these mechanisms underscores their potential as diagnostic and prognostic biomarkers in lung cancer. Methods: In this study, we investigated the diagnostic and prognostic value of a previously validated six-miRNA signature (hsa-miR-26a, hsa-miR-29c, hsa-miR-34a, hsa-miR-30-5p, hsa-miR-30e-3p and hsa-miR-497) in paired tumor and healthy tissue samples from surgically treated NSCLC patients (*n* = 76), who later received platinum-based therapy. To explore the translational applicability of this signature, we also compared miRNA expression levels between matched tumor tissue and plasma samples (n = 36). We further examined the presence of an “inflamed” TME and its association with the miRNA signature by examining the mRNA levels of *TIGIT*, *CD8A*, *FOXP3*, *CXCL10* and *PD-L1*, and performing correlation analysis. Results: All selected miRNAs were significantly downregulated in NSCLC tumor tissue compared to healthy samples. Moreover, miRNA expression was differentially regulated in plasma samples, supporting their potential clinical relevance as minimally invasive biomarkers. The complete six-miRNA signature demonstrated good discriminatory ability between tumor and matched non-malignant tissue (AUC = 0.786, 95% CI: 0.713–0.860; *p* < 0.001), with 72% sensitivity and 77.3% specificity. A significant correlation with relapse-free survival (RFS) (*p* = 0.039) was also observed. Distinct immune-related transcriptional alterations were observed in tumor tissue, including increased expression of immune checkpoint-associated genes (*TIGIT* and *PD-L1*) and reduced *CD8A* expression. Tumor *PD-L1* expression showed modest positive correlations with miR-26a (*p* = 0.046) and miR-30e-3p (*p* = 0.031). Conclusions: Our findings underscore the potential value of this novel six-miRNA signature both as a prognostic and diagnostic biomarker in NSCLC. Its correlation with immune microenvironment characteristics supports its relevance in guiding personalized therapeutic strategies. This signature warrants further clinical validation and may serve as a valuable tool for patient stratification and outcome prediction for patients treated with platinum-based chemotherapy.

## Simple Summary

Patients with non-small-cell lung cancer can respond differently to platinum-based chemotherapy, highlighting the need for reliable biomarkers that can help predict disease outcomes and support treatment decisions. In this study, we evaluated a panel of six small RNA molecules, called microRNAs, in tumor tissue and blood plasma from patients with surgically treated NSCLC who received platinum-based chemotherapy. The six-microRNA signature showed the ability to distinguish tumor from normal lung tissue and was associated with relapse-free survival. The microRNAs were also detectable in plasma, supporting their potential use as minimally invasive blood-based biomarkers. In addition, associations between specific microRNAs and immune-related markers within the tumor were identified, suggesting a possible link between the signature and the tumor immune environment. These findings support further investigation of this six-microRNA signature as a potential tool for patient stratification and outcome prediction in NSCLC.

## 1. Introduction

Lung cancer continues to be a major contributor to cancer-related deaths worldwide, with non-small-cell lung cancer (NSCLC) representing approximately 85% of all lung cancer diagnoses [1]. Tobacco smoking is the major risk factor, although environmental and occupational exposures, genetic susceptibility, and air pollution also contribute to lung carcinogenesis [2]. At the time of diagnosis, around 30–40% of patients with NSCLC have metastatic disease, which is associated with a poor prognosis and a 5-year survival rate below 10% in patients with advanced metastatic NSCLC, primarily due to therapeutic resistance [3,4,5]. Despite advances in diagnosis and therapeutic approaches, NSCLC continues to present a considerable clinical challenge, largely due to late diagnosis, high recurrence rates, variable responses to standard treatments and the aggressive nature of the disease [6,7]. Reliable biomarkers are therefore needed to improve diagnosis, patient stratification, treatment selection, and disease monitoring [8].

Platinum-based chemotherapy remains the cornerstone of treatment for most patients with resectable or advanced NSCLC [9]. Resistance to cisplatin has been attributed to multiple mechanisms, including epigenetic regulation by microRNAs (miRNAs), activation of drug efflux pumps, upregulation of DNA repair pathways and the induction of anti-apoptotic signals [10,11,12]. Among these, the DNA damage response is a key determinant of tumor cell sensitivity to platinum-based chemotherapy. Notably, mutations in DDR-related genes have been linked to tumor metastasis and elevated tumor mutational burden (TMB) in NSCLC [13,14]. These alterations have also been linked to differential responses to both platinum-based chemotherapy and immunotherapy in NSCLC patients, underscoring their significance as biomarkers for treatment stratification [13,14]. However, the heterogeneity in patient responses to platinum-based therapy underlines the urgent need for reliable predictive biomarkers to guide therapeutic decisions and improve clinical outcomes.

miRNAs are small, non-coding RNA molecules, typically 18–25 nucleotides long, that modulate gene expression through post-transcriptional mechanisms. Their biological roles depend on the genes they target, allowing them to act as either tumor suppressors or oncogenes [15]. Their remarkable stability in various biofluids, including blood and urine, highlights their potential as minimally invasive biomarkers for the diagnosis, prognosis and therapeutic monitoring for several pathological conditions, including cancer [15]. However, methodological heterogeneity and the lack of standardized analytical procedures remain important barriers to their clinical implementation [16]. Furthermore, miRNAs have emerged as key regulators of cancer progression, drug resistance and immune modulation within the tumor microenvironment (TME) [17]. Specifically, miRNAs play a vital role in regulating immune system development and function and serve as critical mediators of the crosstalk between tumor cells and the immune microenvironment [18]. In NSCLC, miRNAs have been implicated in modulating a wide range of resistance-related processes, including cell proliferation, apoptosis, invasion, DNA repair, epithelial–mesenchymal transition (EMT), autophagy, hypoxia adaptation, drug efflux, and cancer stemness [19,20,21,22]. Moreover, several miRNAs have already been shown to modulate sensitivity to platinum-based chemotherapy in NSCLC [9,23]. Notably, circulating miRNAs are increasingly recognized as dynamic biomarkers capable of capturing molecular changes during tumor evolution and potentially predicting therapeutic outcomes [24]. In this context, liquid biopsy has emerged as a minimally invasive approach that can complement tissue biopsy by enabling the detection and longitudinal monitoring of tumor-associated molecular alterations. Circulating biomarkers, including circulating tumor DNA (ctDNA), circulating tumor cells (CTCs), extracellular vesicles, and miRNAs, may provide clinically relevant information on tumor evolution and treatment response [25]. However, their prognostic and predictive significance in relation to patient survival and treatment response remains largely unexplored.

Building on our previous work, which identified a six-miRNA signature (miR-26a, miR-29c, miR-34a, miR-497, miR-30e-3p and miR-30e-5p) associated with response to platinum-based chemotherapy in NSCLC [26], the present study aimed to further investigate its clinical relevance in a larger cohort of patients with resected NSCLC who received adjuvant platinum-based chemotherapy. Specifically, we evaluated the diagnostic and prognostic potential of the six-miRNA signature in tumor tissue and assessed its expression in paired plasma samples to explore its potential as a minimally invasive biomarker. We further examined the association between these miRNAs and features of the TME, considering the emerging link between DDR, chemotherapy-induced inflammation, and antitumor immune activation. By integrating molecular and immunological parameters, this study aimed to further assess the clinical applicability of miRNA-based biomarkers in NSCLC, with potential implications for patient stratification, treatment decision-making, and the development of more personalized therapeutic approaches.

## 2. Materials and Methods

### 2.1. Patients Characteristics

The study cohort included 76 patients (n = 76) diagnosed with operable NSCLC between 2007 and 2017 at the Department of Medical Oncology, University General Hospital of Heraklion, Crete. Matched non-malignant tissue specimens were available for all patients. In addition, paired plasma samples were available from 36 patients (n = 36) and were collected following surgical resection of the primary tumor but prior to the initiation of adjuvant chemotherapy. Patients who had received neoadjuvant therapy were excluded. Archived formalin-fixed, paraffin-embedded (FFPE) tissue specimens were obtained from the Department of Pathology at the University General Hospital of Heraklion and independently evaluated by two pathologists. Plasma samples exhibiting a pinkish discoloration, suggestive of hemolysis, or with inadequate RNA quality, were excluded from subsequent analyses. Clinicopathological data and follow-up information were prospectively collected for each patient and are summarized in Table S1. Written informed consent was obtained from all participants, and the study received approval from the Ethics and Scientific Committee of the University General Hospital of Heraklion (approval ID: 7696; Crete, Greece).

### 2.2. Blood Sample Collection

Peripheral blood samples were obtained from patients with NSCLC early in the morning hours and collected into ethylenediaminetetraacetic acid (EDTA) tubes. Plasma separation was performed within 2 h of collection by centrifuging the samples at 2500 rpm for 15 min at 4 °C. A second centrifugation step at 2000× *g* for 15 min at 4 °C was then carried out to eliminate residual cellular debris. The resulting plasma was divided into aliquots and stored at −80 °C until further analysis.

### 2.3. Total RNA Isolation from FFPE Tissue and Plasma Samples

All FFPE tumor specimens were independently examined by two pathologists to confirm specimen suitability and identify the most representative tumor regions for microdissection. The corresponding non-malignant tissue specimens were also evaluated. Serial sections measuring 5 μm in thickness were prepared from each selected paraffin block and stained with hematoxylin–eosin. Malignant cells were subsequently isolated using a piezoelectric microdissector (Eppendorf, Hamburg, Germany). For each sample, 10,000 cells were incubated overnight at 56 °C in 400 μL of lysis buffer containing 1.5 mg/mL proteinase K [27]. On the following day, total RNA was extracted using TRIzol LS (Ambion, Life Technologies, Austin, Texas, USA). Following denaturation, 25 fmol of synthetic Caenorhabditis elegans miRNA cel-miR-39 (Qiagen Inc., Germantown, Maryland, USA) was added as an exogenous normalization control [21,28]. The resulting RNA pellets were dissolved in 30 μL of RNase-free water and treated with DNase (Thermo Scientific, Waltham, MA, USA) to remove residual genomic DNA contamination.

Plasma specimens were thawed on ice and centrifuged at 14,000 rpm for 15 min to eliminate residual cellular debris. Total RNA was subsequently isolated from 400 μL of plasma using TRIzol LS (Ambion, Life Technologies), following a previously established protocol [29]. Following denaturation, 5 μL containing 25 fmol of synthetic *C. elegans* miRNA cel-miR-39 (Qiagen Inc., Hilden, Germany) was introduced as an exogenous control. Phase separation was achieved by adding 300 μL of chloroform, followed by a 10 min incubation on ice and centrifugation at 14,000 rpm for 15 min at 4 °C. The resulting aqueous phase (700 μL) was transferred into Eppendorf tubes, and RNA precipitation was performed by adding 0.7 volumes of isopropanol and 1 μL of glycogen (final concentration: 13 μg/mL; QIAGEN GmbH, Hilden, Germany). Samples were then incubated overnight at −80 °C. The RNA pellets were washed three times with 75% ethanol, allowed to air-dry, and dissolved in 50 μL of RNase-free water. RNA concentration and purity were determined for both FFPE-derived and plasma-derived samples using a NanoDrop 1000 Spectrophotometer (Thermo Fisher Scientific, Waltham, MA, USA). All RNA samples were aliquoted and maintained at −80 °C until subsequent analysis.

### 2.4. RT-qPCR Analysis and miRNA/mRNA Expression

Complementary DNA (cDNA) synthesis and quantitative real-time PCR (RT-qPCR) were performed using TaqMan technology in accordance with the manufacturer’s protocols, as previously reported [30]. Briefly, 20 ng of total RNA isolated from tissue specimens was reverse-transcribed in a final reaction volume of 5 μL using the TaqMan MicroRNA Reverse Transcription Kit and miRNA-specific stem-loop primers (Applied Biosystems, Foster City, CA, USA; Table S2). For plasma-derived RNA, 10 ng of total RNA was used per reverse transcription reaction. Reverse transcription was conducted in a Peltier Thermal Cycler PTC-200 under the following conditions: 30 min at 16 °C, 30 min at 42 °C, and 5 min at 85 °C.

Following reverse transcription, the cDNA was diluted to a final volume of 20 μL. Each miRNA was subsequently quantified in a 5 μL PCR reaction containing 1× TaqMan Universal PCR Master Mix (2×, No AmpErase UNG), 0.25 μL of the corresponding TaqMan MicroRNA Assay, and 2.25 μL of diluted cDNA. Amplification was performed using a ViiA 7 Real-Time PCR System (Applied Biosystems, Foster City, CA, USA), with an initial incubation at 95 °C for 10 min, followed by cycling at 95 °C for 15 s and 60 °C for 1 min. All reactions were carried out in triplicates. Negative controls were included during both reverse transcription and RT-qPCR, with RNA replaced by H_2_O in the reverse transcription reactions and no-template controls included in the PCR assays. Synthetic C. elegans miRNA cel-miR-39 served as an exogenous reference to monitor technical variability associated with RNA isolation and reverse transcription efficiency. Relative miRNA expression was determined using the 2^−ΔCt method, using sample type-specific endogenous reference controls. For FFPE tissue samples, miR-1228-3p was used as the endogenous reference and ΔCt was calculated as Ct_target miRNA − Ct_miR-1228-3p. For plasma samples, U6 snRNA was used as the endogenous reference and ΔCt was calculated as Ct_target miRNA − Ct_U6 snRNA [31,32]. Although miR-1228 has previously been proposed as a reference for circulating miRNA quantification, its suitability for normalization in FFPE tissue specimens was also evaluated in the present study. No significant difference in miR-1228 expression was detected between tumor specimens and their matched non-malignant counterparts (Wilcoxon signed-rank test, *p* = 0.142). Furthermore, its expression levels were comparable to those of the target miRNAs, as reflected by the low ΔCt values (ΔCt = Ct_target − Ct_reference). U6 snRNA was additionally selected as an endogenous reference based on its stable and reproducible expression across the patient cohort (Mann–Whitney test, *p* = 0.272). The predefined acceptable mean cycle threshold (Ct) ranges were 30 < Ct < 33 for both miR-1228 and U6 snRNA, and 20 < Ct < 22 for cel-miR-39. Samples exhibiting a mean Ct value greater than 35, no detectable amplification of the target miRNAs, or reference Ct values outside the established acceptable ranges, potentially indicating suboptimal RNA extraction, were excluded from subsequent statistical analyses (n = 1 for miR-1228 and n = 3 for U6 snRNA).

cDNA was synthesized from 300 ng of total RNA using the SuperScript™ III First-Strand Synthesis SuperMix (Invitrogen, Carlsbad, CA, USA). Each 20 μL reverse transcription reaction contained 10 μL of 2× RT Reaction Mix, 2 μL of RT Enzyme Mix, the required volume of RNA, and DEPC-treated water to reach the final volume. Reverse transcription was performed under the following conditions: 25 °C for 10 min, 50 °C for 30 min, and 85 °C for 5 min. Subsequently, 1 μL of RNase H was added to each reaction, followed by incubation at 37 °C for 20 min to degrade the RNA strand of the RNA–cDNA hybrids and obtain single-stranded cDNA. RT-qPCR was performed using 1 μL of cDNA template derived from an initial RNA input of 300 ng, 6.25 μL of TaqMan Universal Master Mix (Applied Biosystems), and gene-specific primers and probes (Table S3). DEPC-treated water was added to obtain a final reaction volume of 11 μL. The amplification protocol consisted of an initial incubation at 95 °C for 10 min, followed by 45 cycles of 95 °C for 15 s and 60 °C for 1 min.

The relative mRNA expression levels of *TIGIT*, *STING*, *CD8A*, *FOXP3*, *CXCL10*, and *PD-L1* were assessed using the ViiA 7 Real-Time PCR System (Applied Biosystems, Foster City, CA, USA), with β-actin serving as the endogenous reference gene. The primer and probe sequences for β-actin were designed using Primer Express 2.0 Software (Applied Biosystems), whereas the TaqMan 20× probes were obtained from Applied Biosystems (Foster City, CA, USA). All assays were conducted in triplicate. To assess potential genomic DNA contamination, each RNA sample was also analyzed using a control reaction in which the reverse transcription step was omitted.

### 2.5. Statistical Analysis

Statistical analyses were conducted using SPSS version 29.0.2.0 (SPSS Inc., Chicago, IL, USA; accessed on 31 March 2025) and GraphPad Prism version 9.3.1 (GraphPad Software Inc., La Jolla, CA, USA). Differences in miRNA expression between matched tumor tissue and plasma samples were evaluated using the non-parametric Wilcoxon signed-rank test, while correlations between tissue and plasma expression levels were examined using Spearman’s rank correlation coefficient.

Patients were stratified into high- and low-expression groups based on the median expression value of each miRNA or mRNA. Expression levels equal to or greater than the median were classified as high, whereas values below the median were classified as low. Associations between miRNA expression and clinicopathological characteristics were assessed using the chi-squared test. Differences in clinicopathological variables across NSCLC sample groups were examined using the Mann–Whitney U, Jonckheere–Terpstra, or Kruskal–Wallis test, as appropriate.

RFS and overall survival (OS) were analyzed using the Kaplan–Meier method, with differences between survival curves assessed by the log-rank test. Univariate and multivariable Cox proportional hazards regression models were applied to investigate the prognostic significance of the 6-miRNA signature, immune-related genes, and clinical parameters for the respective survival endpoints. The full multivariable model incorporated established prognostic indicators and clinically relevant factors (age, stage, grade, smoking status, sex and lymph node status) for NSCLC. Hazard ratios (HRs) and their corresponding 95% confidence intervals (CIs) were calculated.

OS was defined as the interval from diagnosis or surgery to death or the most recent follow-up, whereas RFS was measured from the initiation of adjuvant treatment to the first documented disease progression. Patients who remained alive or had not experienced disease progression at the time of analysis were censored at their last follow-up.

Receiver operating characteristic (ROC) curves were generated, and the area under the curve (AUC) was calculated to assess the diagnostic performance of each miRNA. The optimal cutoff for each miRNA was identified by maximizing Youden’s index (sensitivity + specificity − 1), and the corresponding sensitivity and specificity were determined. All statistical tests were two-sided, with *p* < 0.05 considered statistically significant. The manuscript was prepared in accordance with the Reporting Recommendations for Tumor Marker Prognostic Studies (REMARK) guidelines [33].

## 3. Results

**Figure 1:**
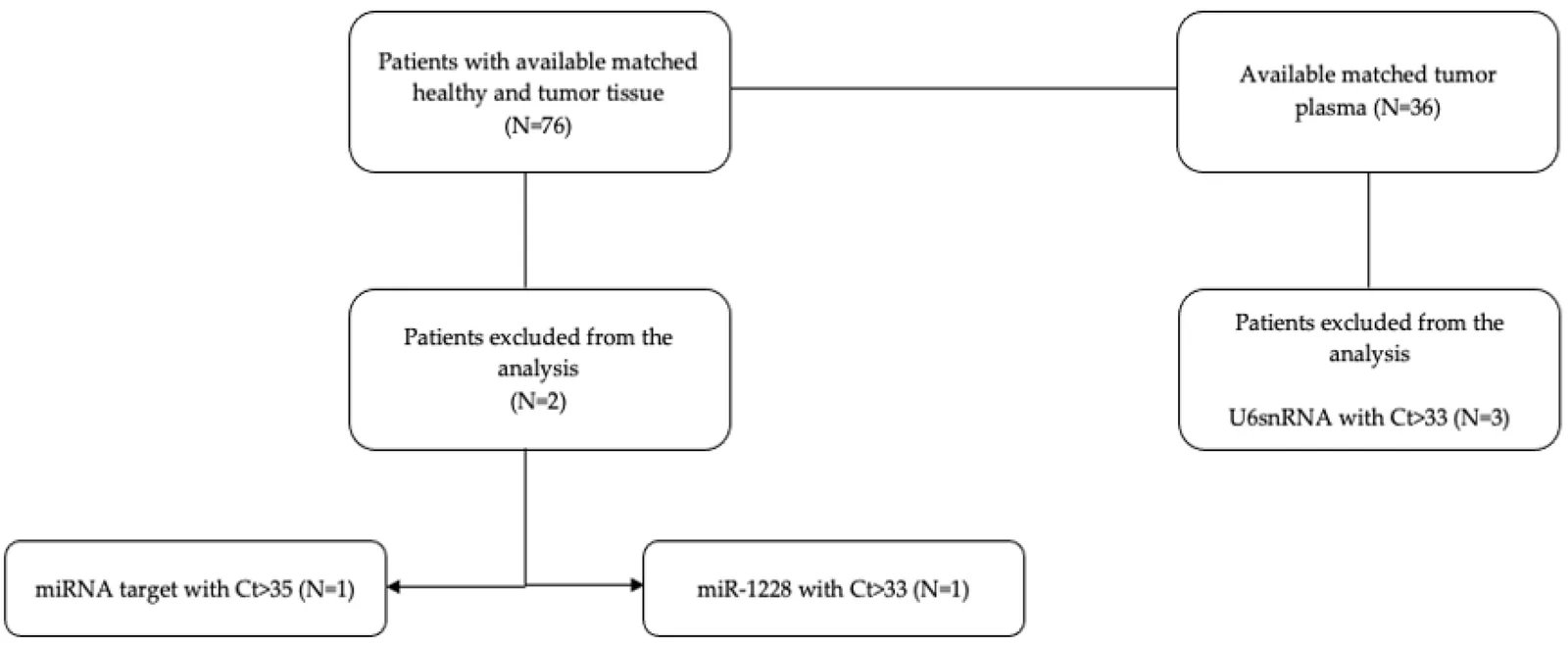
Flowchart of the study.

The selection of the six-miRNA signature panel (miR-26a, miR-29c, miR-34a, miR-497, miR-30e-3p, and miR-30e-5p) was based on findings from our previously published in silico study [26]. This prior analysis in a small cohort of approximately 20 patients identified these miRNAs as potential diagnostic and prognostic biomarkers for NSCLC and possible indicators of NSCLC patients’ response to cisplatin, providing the rationale for their inclusion in the present study (Figure 1).

### 3.1. Patient Characteristics and Study Design

Patient characteristics and flow of the study are depicted in Table S1 and Figure 1, respectively. A total of 72.4% of the patients were less than 70 years old; 86.8% of the patients were male; 57.8% of the patients had non-Squamous Cell Carcinoma (SqCC) histologic type; and 51.3% of the patients experienced relapse.

### 3.2. Relative Expression Levels of the Selected miRNAs in Paired Tumor and Healthy Tissue Samples and Their Discriminatory Capacity

Expanding the cohort size of our previously published study [26], the expression levels of the six miRNAs were evaluated in primary tumor tissues and paired adjacent normal tissues using RT-qPCR. All six miRNAs showed statistically significant differential expression between tumor and normal tissues. Specifically, all the selected miRNAs showed marked downregulation in tumor vs. normal tissues, with strong statistical significance (miR-26a, miR-29c, miR-497, miR-30e-3p and miR-30e-5p, *p* ≤ 0.0001; miR-34a, *p* ≤ 0.05). These consistent and significant differences highlight a coordinated deregulation of the six-miRNA signature in tumor tissue, supporting its potential relevance in tumor-associated molecular alterations.

**Figure 2:**
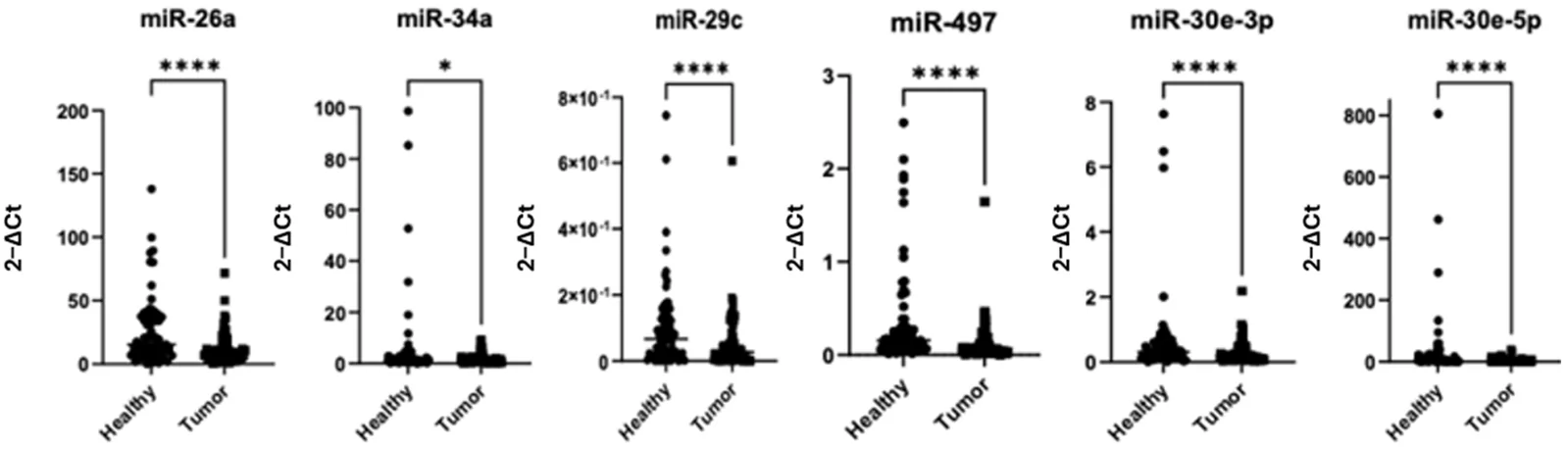
Relative expression of the six miRNAs in primary tumor specimens and matched non-malignant tissues. Differences in the expression of each miRNA between paired tumor and normal tissue samples were evaluated using the non-parametric Wilcoxon signed-rank test. The Y-axis represents miRNA expression normalized to miR-1228, calculated using the 2^ − ΔCt method. Exact *p*-values are shown (* *p* ≤ 0.05; **** *p* ≤ 0.0001).

Spearman’s rank correlation analysis demonstrated predominantly moderate-to-strong positive correlations among the majority of the six miRNAs within tumor tissue (all *p* < 0.001). miR-26a and miR-34a showed robust associations with multiple members of the signature, including miR-29c, miR-497, miR-30e-3p, and miR-30e-5p. Strong correlations were also observed between miR-34a and miR-30e-3p, as well as between miR-29c and miR-497. In contrast, no significant correlation was detected between miR-29c and miR-30e-5p (Rho = 0.078, *p* = 0.507) (Figure 2).

**Figure 3:**
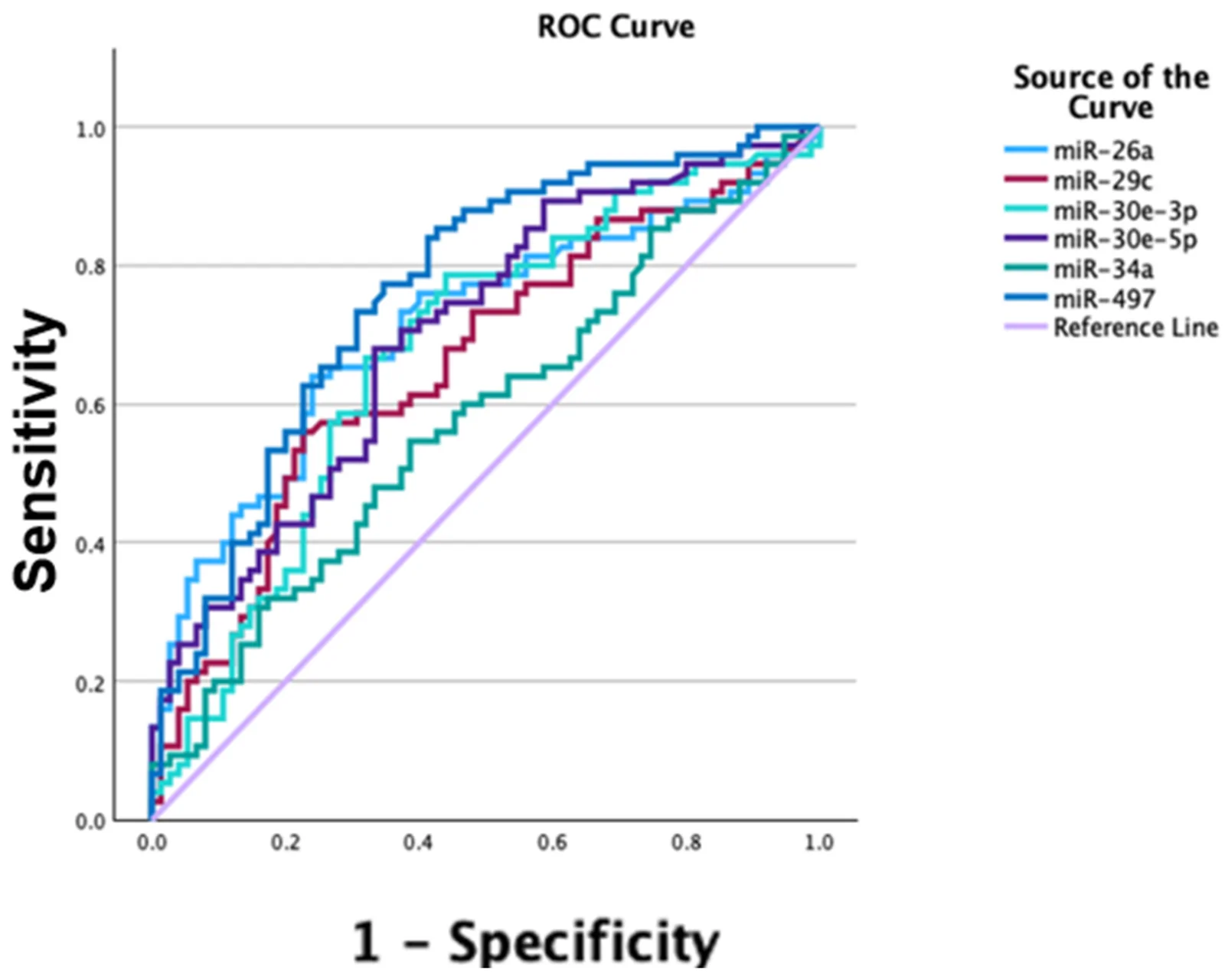
ROC analysis of all individual miRNAs.

Furthermore, in order to assess the ability of individual miRNAs to discriminate tumor from healthy tissues, ROC curve analysis was performed, followed by calculation of AUCs and Youden indices. Among the evaluated markers, miR-497 demonstrated the highest diagnostic potential with an AUC of 0.762 (95% CI: 0.686–0.839, *p* < 0.001), sensitivity of 85.3% and specificity of 57.3%. miR-26a (AUC = 0.713, 95% CI: 0.629–0.796, *p* < 0.001) and miR-30e-5p (AUC = 0.702, 95% CI: 0.619–0.785, *p* < 0.001) followed with sensitivities of 64.0% and 68.0%, respectively, and specificities of 76.0% and 66.7%, respectively. miR-30e-3p and miR-29c exhibited moderate diagnostic ability (AUCs of 0.676 and 0.661, respectively; both *p* < 0.001) with sensitivity of 78.7% and 56.0% respectively, and specificity of 56.0% and 77.3%, respectively. In contrast, miR-34a showed limited diagnostic value and did not reach statistical significance (AUC = 0.579, *p* = 0.089) with sensitivity of 54.7% and specificity of 61.3% (Figure 3).

**Figure 4:**
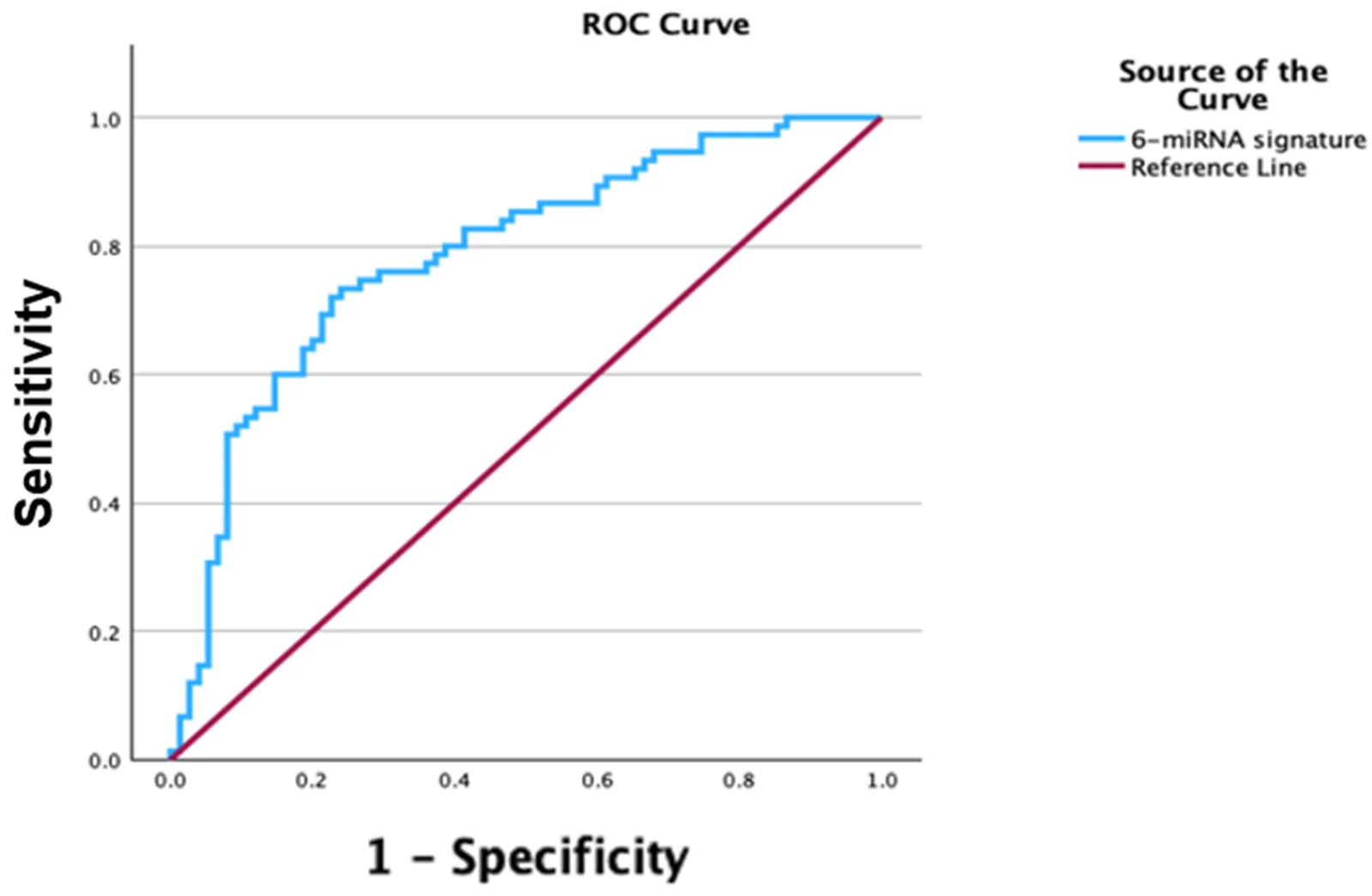
ROC analysis for the complete 6-miRNA signature (miR-26a, miR-34a, miR-29c, miR-497, miR-30e-3p and miR-30e-5p).

ROC curve analysis was also performed on the combined six-miRNA signature using the predicted probabilities derived from logistic regression. The complete signature demonstrated good discriminatory ability between tumor and matched non-malignant tissue, with an AUC of 0.786 (95% CI: 0.713–0.860, *p* < 0.001). Using the Youden index reminder, the optimal cutoff for the six-miRNA signature was identified at a predicted probability of 0.585, yielding a sensitivity of 72.0% and a specificity of 77.3% for discriminating tumor from healthy tissues (Figure 4).

### 3.3. miRNA Expression and Patient Clinicopathological Characteristics

We further evaluated the association between tissue miRNA expression and different patient clinicopathological characteristics (gender, age, grade, stage, tumor size, relapse status, histotype, smoking status and lymph node status). Specifically, miR-29c expression in tumor tissues differed significantly between sexes, with higher levels observed in female compared with male patients (Mann–Whitney U test, *p* = 0.047), whereas no significant sex-based differences were detected for the remaining miRNAs of the six-miRNA signature.

miR-30e-5p expression demonstrated a significant stage-dependent trend across pathological stages (Jonckheere–Terpstra test, *p* < 0.001), whereas no significant associations with tumor stage were observed for the other miRNAs. Pairwise comparisons further indicated that differences in miR-30e-5p expression were mainly driven by contrasts between early-stage (IA/IB) and more advanced stages (IIIA/IIIB), supporting a stage-dependent increase in expression. Moreover, miR-30e-5p expression differed significantly according to smoking status (Kruskal–Wallis test, *p* = 0.004), with post hoc analyses indicating that the difference was primarily driven by contrasts between non-smokers and former smokers, with non-smokers presenting lower levels of miR-30e-5p. No smoking-related differences were observed for the remaining miRNAs. miR-30e-5p expression also differed significantly according to relapse status (Kruskal–Wallis test, *p* = 0.038), showing higher tumor expression in relapsed patients.

Finally, expression levels of miR-26a, miR-34a, miR-30e-3p and miR-30e-5p differed significantly according to lymph node status (*p* = 0.009, *p* = 0.004, *p* = 0.022 and *p* < 0.001 respectively), with higher levels observed in LN-positive tumors, whereas miR-29c and miR-497 showed no association with nodal involvement (*p* = 0.189 and *p* = 0.096, respectively).

### 3.4. Differential Tissue–Plasma Expression and Correlation Analysis of the Six-miRNA Signature

**Figure 5:**
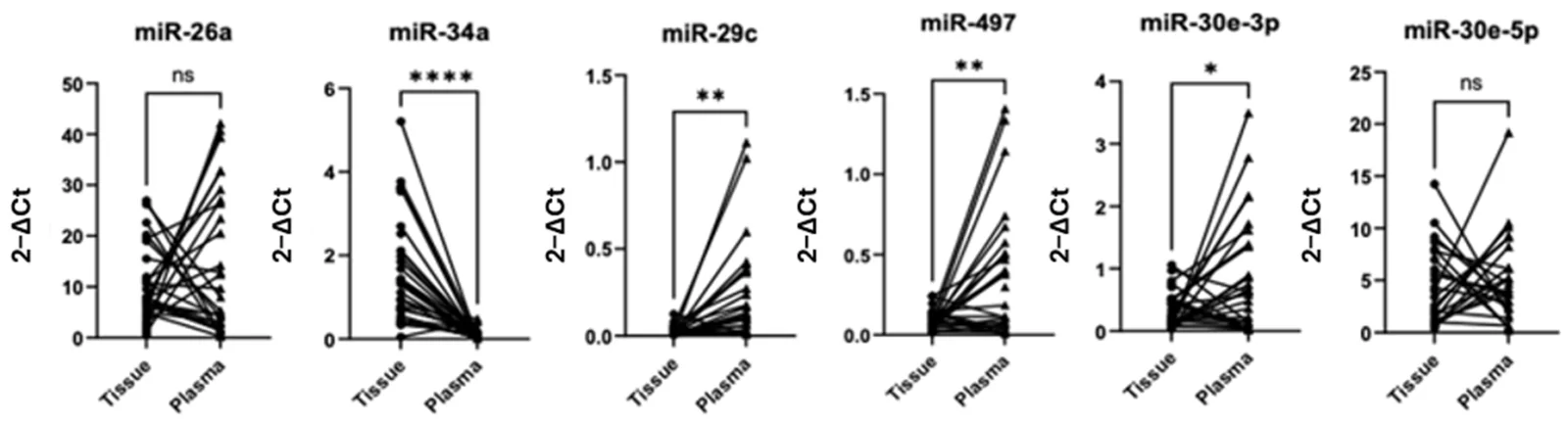
Relative expression levels of the 6-miRNA signature in tumor tissue samples and their matched plasma samples (n = 36). Comparison for each miRNA was performed by non-parametric Wilcoxon paired sample test. The Y-axis denotes expression levels for each miRNA calculated using the 2^−ΔCt method, with miR-1228-3p used as the endogenous reference for tissue samples and U6 snRNA used as the endogenous reference for plasma samples. Exact *p* values are indicated (* *p* ≤ 0.05; ** *p* ≤ 0.01; **** *p* ≤ 0.0001).

The relative expression levels of the six miRNAs (miR-26, miR-34a, miR-29c, miR-497, miR-30e-3p, and miR-30e-5p) were evaluated in tumor tissue and available matched plasma samples from NSCLC patients (n = 36). Comparative analysis using the non-parametric Wilcoxon paired sample test revealed differential expression patterns between tissue and plasma. Specifically, miR-34a expression levels were significantly downregulated (*p* ≤ 0.0001) in plasma samples compared to tumor tissue, whereas miR-29c, miR-497 and miR-30e-3p showed significant upregulation in plasma vs. tumor tissues (miR-29c and miR-497, *p* ≤ 0.01; miR-30e-3p, *p* ≤ 0.05). In contrast, miR-26 and miR-30e-5p did not exhibit significant differences between tissue and plasma (Figure 5). Correlation analysis using Spearman’s rank test revealed a moderate but significant inverse association between tumor and plasma expression levels of miR-34a (Rho = −0.372, *p* = 0.025), whereas no significant correlations were identified for miR-26a (Rho = 0.126, *p* = 0.464), miR-29c (Rho = −0.265, *p* = 0.118), miR-497 (Rho = −0.030, *p* = 0.862), miR-30e-3p (Rho = −0.121, *p* = 0.483) and miR-30e-5p (Rho = −0.076, *p* = 0.483). These findings indicate that selected miRNAs from our signature are detectably and differentially represented in circulation, supporting their potential utility as circulating, minimally invasive biomarkers in NSCLC. Specifically, for miR-34a, higher intratumoral levels are associated with lower circulating levels, suggesting a dynamic regulation of miR-34a between tumor tissue and circulation.

### 3.5. Circulating miRNAs and Patients’ Characteristics

Similar to tissue miRNA, we sought to identify associations between plasma miRNA expression and clinicopathological characteristics. No significant associations were uncovered for gender, age at diagnosis, histological subtype, smoking status, tumor grade, tumor size, or lymph node status (all *p* > 0.05). Conversely, pathological stage was significantly associated with the plasma expression of miR-30e-3p (*p* = 0.023), miR-30e-5p (*p* = 0.040), miR-26a (*p* = 0.017), miR-34a (*p* = 0.037), and miR-29c (*p* = 0.027), whereas no significant association was observed for miR-497 (*p* = 0.085).

### 3.6. Differential Expression and Correlation Analysis of Immune-Related mRNAs in Tumor Versus Healthy Tissue Samples

**Figure 6:**
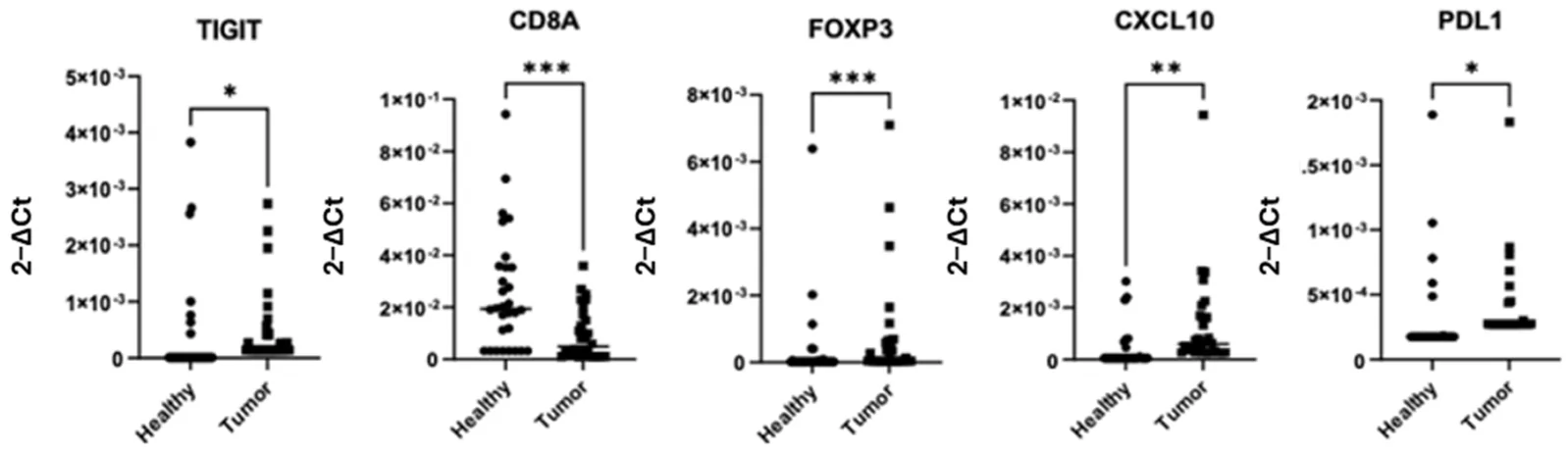
Relative mRNA levels of TIGIT, CD8A, FOXP3, CXCL10 and PD-L1 in NSCLC tumor tissue versus paired healthy lung tissue samples measured by RT-qPCR. Expression values were normalized to β-actin and reported as 2^−ΔCT^. For mRNA expression analyses, a subset of samples (n = 30) from the same cohort was included, based on RNA quality and availability criteria required for reliable downstream analysis. Statistical significance was assessed with non-parametric Wilcoxon paired sample test. Exact *p* values are indicated (* *p* ≤ 0.05; ** *p* ≤ 0.01; *** *p* ≤ 0.001).

The relative mRNA expression levels of the immune-related genes *TIGIT*, *CD8A*, *FOXP3*, *CXCL10*, and *PD-L1* were analyzed in NSCLC tumor tissues and matched adjacent healthy lung tissues using RT-qPCR. Tumor tissues exhibited significantly altered expression profiles compared with paired healthy regions. Specifically, *TIGIT*, *FOXP3*, *CXCL10*, and *PD-L1* mRNA expression levels were significantly upregulated in tumor tissues (*TIGIT*, *PD-L1 p* ≤ 0.05; *FOXP3 p* ≤ 0.001; *CXCL10 p* ≤ 0.01), indicating enhanced immune checkpoint signaling and immune cell infiltration, such as T-cells, NK cells and macrophages within the TME. In contrast, *CD8A* expression, a marker of cytotoxic T-lymphocytes, was significantly reduced (*p* ≤ 0.001) in tumor samples compared with normal lung tissue, suggesting reduced CD8^+^ T-cell numbers (Figure 6). These findings highlight distinct immune-related transcriptional alterations in NSCLC tumors relative to normal lung tissue.

Correlation analysis revealed significant positive associations among immune-related transcripts. *CD8A* expression showed a moderate positive correlation with *CXCL10* (Rho = 0.334, *p* = 0.003) and PD-L1 (Rho = 0.258, *p* = 0.024), while *FOXP3* expression correlated positively with *CXCL10* (Rho = 0.304, *p* = 0.008) and *PD-L1* (Rho = 0.328, *p* = 0.004). In addition to correlations among immune cell-associated markers, significant positive associations were also observed between immune signaling and immune checkpoint-related genes (PD-L1, TIGIT). *CXCL10* expression correlated positively with *PD-L1* (Rho = 0.301, *p* = 0.008), while *PD-L1* also showed a significant positive correlation with *TIGIT* (Rho = 0.233, *p* = 0.043). These associations indicate coordinated expression of immune chemokines and immune checkpoint molecules, suggesting a linked activation of immune signaling and immune regulatory pathways within the analyzed samples.

Interestingly, tumor PD-L1 expression positively correlated with miR-26a (Rho = 0.231, *p* = 0.046) and miR-30e-3p (Rho = 0.249, *p* = 0.031) expression, suggesting a potential link between specific components of the miRNA signature and immune checkpoint regulation within the TME. While the observed associations are modest, they may indicate that these miRNAs are involved in pathways related to immune evasion or inflammatory signaling rather than reflecting a global immune activation pattern. No other correlations were observed between the selected miRNAs and immure-related genes.

### 3.7. Immune-Related mRNAs and Patient Clinicopathological Characteristics

Similar to our miRNA analyses, we evaluated the correlation between different patient clinicopathological characteristics (gender, age, grade, stage, tumor size, relapse status, histotype, smoking status and lymph node status) and expression levels of immune-related mRNAs. Post hoc pairwise comparisons indicated that CD8A expression was higher in stage IIB patients compared to stage IIA (*p* = 0.027). Moreover, STING and CD8A expression levels were higher in ADC subtype patients in comparison to SqCC histology (*p* = 0.032, *p* = 0.029, respectively). Additionally, CD8A, FOXP3 and CXCL10 levels were lower in former smokers than in active smokers (*p* = 0.026, *p* = 0.011 and *p* = 0.012, respectively). Higher levels of *PD-L1* expression were also observed in tumors ≤ 3 cm and progressively decreased in intermediate (3–7 cm) and large (>7 cm) tumors (*p* = 0.047).

### 3.8. miRNA Expression and Survival Outcomes

In order to evaluate the prognostic relevance of the examined miRNA expression in terms of RFS and OS, a stratified Kaplan–Meier survival curve analysis was performed. We did not observe any significant associations between individual miRNA expression and survival, whether in tissue or in plasma (Supplementary Figures S1–S4).

To further evaluate the prognostic significance of the miRNAs, univariate and multivariate analyses were performed based on the expression levels of the six miRNAs (classified into high or low). Univariate Cox regression analysis revealed that none of the examined miRNAs were significantly associated with RFS or OS (Table 1). However, the multivariable Cox regression model incorporating all six miRNAs was statistically significant for RFS *(p* = 0.039, *χ*^2^ = 13.283), indicating that the combined miRNA expression signature significantly relates to the RFS status. Regarding individual miRNAs, reduced expression of miR-26a (HR = 2.78, *p* = 0.027, 95% CI: 1.121–6.877), miR-29c (HR = 2.89, *p* = 0.020, 95% CI: 1.185–7.049) and miR-30e-5p (HR = 3.08, *p* = 0.036, 95% CI: 1.073–8.837) was independently associated with an increased hazard or relapse, whereas low expression of miR-34a was independently associated with a reduced risk of relapse (HR = 0.248, *p* = 0.008, 95% CI: 0.089–0.694). miR-497 (HR = 0.537, *p* = 0.158, 95% CI: 0.226–1.274) and miR-30e-3p (HR = 0.797, *p* = 0.640, 95% CI: 0.308–2.063) were not independently associated with RFS.

Multivariable analysis was also performed for OS. The overall miRNA signature was not significantly associated with OS s (*χ*^2^ = 7.628, *p* = 0.267). Only mir-30e-5p was independently associated with OS (HR = 10.293, *p* = 0.029, 95% CI: 1.274–83.154) (Table 1).

## 4. Discussion

NSCLC exhibits significant interpatient variability, highlighting the critical need for reliable biomarkers to guide patient stratification and optimize clinical outcomes [12]. Previous research has drawn increasing attention to miRNAs as regulators of key cancer-related processes, including DDR, cell survival, cancer invasion, metastasis and angiogenesis, which positions them at the intersection of tumor biology and therapeutic sensitivity [34]. Beyond their biological relevance, miRNAs are detectable in both solid and liquid biopsy samples, reinforcing their clinical utility in a minimally invasive setting [35]. In fact, miRNAs are considered highly stable in peripheral plasma because of their small size and relative nuclease resistance [36,37].

Building on our previous in silico study that identified a core six-miRNA panel with potential predictive value for cisplatin response, the present study investigates the clinical relevance of this signature in resectable NSCLC. By analyzing miRNA expressions in matched tumor and adjacent normal tissues from surgically resected NSCLC patients receiving platinum-based chemotherapy, as well as matched plasma-derived samples, we aimed to clarify their potential as diagnostic and prognostic tools in early-stage disease. In the current study, we showed that all six miRNAs were downregulated in tumor vs. matched normal tissues, supporting their diagnostic potential. Importantly, combining all six miRNAs improved the overall discriminatory performance, yielding an AUC of 0.786 with 72% sensitivity and 77.3% specificity for distinguishing tumor from matched non-malignant tissues. These findings suggest that a multi-miRNA signature may provide greater diagnostic value than individual miRNAs alone. These findings are consistent with previous studies reporting that miRNA-based signatures can discriminate patients with lung cancer from non-cancer controls [38]. Moreover, all six miRNAs were consistently detected in matched plasma samples, with four of them (miR-29c, miR-497, miR-30e-3p and miR-34a) displaying significantly different levels between tumor tissues and matched plasma samples. This highlights their stability in circulation and potential utility as minimally invasive biomarkers. Importantly, the expression of the total six-miRNA panel as well as of certain miRNAs (miR-26a, miR-29c, miR-30e-5p and miR-34a) of this panel was significantly associated with clinical outcomes (RFS), underscoring their prognostic value in the context of platinum-based chemotherapy.

Emerging evidence indicates that key components of the DNA repair machinery are subject to epigenetic regulation by miRNAs [39,40]. Moreover, numerous studies have linked specific miRNAs to sensitivity or resistance to a broad range of anticancer agents, including cisplatin. Specifically, for miR-26a, where the majority of evidence points towards a tumor-suppressing role in NSCLC [41,42], elevated miR-26a expression has been shown to sensitize lung adenocarcinoma cancer and cancer stem cells to the cytotoxic effects of cisplatin and paclitaxel, respectively [43,44]. These findings are consistent with our previous study, in which miR-26a was identified as a potential predictive biomarker of response to platinum-based chemotherapy, further supporting its relevance in NSCLC treatment response [26]. Interestingly, plasma miR-26a levels were found to increase in patients with NSCLC [45], indicating the possibility that cancer cells may release miR-26a into the circulation. Our group has previously shown that high plasma miR-26a levels correlated with shorter OS in patients with advanced SqCC receiving first-line platinum-based chemotherapy [28]. In the current study, which included patients with early-stage, resectable NSCLC, high tissue miR-26a expression correlated with higher RFS. These results are in keeping with the potential role of miR-26a (measured in tissue or plasma) both as a prognostic and treatment-predictive biomarker in NSCLC patients, whether in early or in advanced disease stage.

Regarding miR-30e, several studies indicate that members of this family (-3p and -5p) may have tumor-suppressive activities in various malignancies [46,47]. miR-30e-5p has been shown to inhibit cell proliferation and invasion in NSCLC by directly targeting SOX9, a transcription factor involved in tumorigenesis and metastasis [48]. Interestingly, lower miR-30e-5p expression has been found in NSCLC tumor tissue and plasma samples compared to healthy controls [49,50,51]. Furthermore, high circulating (but not tissue) miR-30e-5p levels were found to be associated with shorter OS in surgically resected NSCLC patients [49]. Our findings are consistent with previously published data as miR-30e-5p levels were reduced in tumor tissues, while at the same time increased miR-30e-5p expression showed a protective survival effect, providing further evidence for the relevance of this miRNA in NSCLC progression and treatment response. Moreover, we observed that non-smokers exhibited lower levels of miR-30e-5p, a finding that is consistent with previous reports by Huang et al. (2025) [52]. This observation may reflect smoking-related alterations in gene regulation, as tobacco exposure has been shown to modulate miRNA expression through inflammatory pathways, oxidative stress, and epigenetic modifications [53].

miR-34a is a tumor suppressor in lung cancer [54,55,56], regulating several biological processes including cellular homeostasis, cell migration, invasion, proliferation, genomic integrity as well as EMT, stemness and chemoresistance [57,58,59,60]. Interestingly, miR-34a is significantly overexpressed in NSCLC patients receiving platinum-based chemotherapy, as cisplatin has been shown to induce its expression. Elevated miR-34a plasma levels have been observed in platinum-sensitive NSCLC patients compared to those with chemoresistant disease [61,62]. Furthermore, reduced tissue levels of miR-34a have been associated with increased rate of relapse and poor prognosis in NSCLC patients who underwent curative surgery [61]. Similarly, low plasma miR-34a has been correlated with reduced disease-free survival and OS in NSCLC patients [55]. In our analysis, high tissue miR-34a levels did not show a protective survival effect. Compared to the cohort of Gallardo et al., our patients had overall higher disease stage (stage II–III: ~80% vs. 45%); however, the association of stage with the discrepancy observed between the two studies remains speculative [61]. The inverse correlation observed in our study between tissue and plasma miR-34a levels may reflect differences in intracellular retention, selective release into the circulation, extracellular vesicle-mediated transport, or differential extracellular stability and clearance. However, the present study did not investigate the mechanisms underlying miR-34a release, and these potential explanations therefore remain speculative. More broadly, the limited concordance observed between tissue and circulating miRNA levels highlights that plasma miRNA abundance may not directly reflect intratumoral expression.

Across multiple malignancies, members of the miR-29 family (miR-29a/b/c) generally exhibit tumor-suppressive activity and are frequently downregulated [63,64]. For example, overexpression of miR-29c has been shown to enhance cisplatin sensitivity, whereas its downregulation is associated with cisplatin resistance in lung cancer cells, suggesting a potential positive correlation between miR-29c expression and cisplatin response [65]. In NSCLC specifically, miR-29c is downregulated in tumor tissue, consistent with our findings demonstrating significantly reduced miR-29c expression in tumor tissue samples. Restoration of miR-29c can decrease cisplatin resistance in NSCLC cell lines, while at the same time higher expression of miR-29c has been associated with longer disease-free survival and improved outcomes in NSCLC patients receiving adjuvant cisplatin chemotherapy [65]. In our cohort, increased tumor miR-29c expression correlated with increased RFS. A recent study also showed that plasma exosomal miR-29c-5p can distinguish lung adenocarcinoma from benign lung disease patients [66].

Finally, growing evidence indicates that miR-497 is consistently downregulated across various malignancies, including NSCLC [67,68]. Interestingly, reduced miR-497 is observed both in NSCLC tumor tissue and plasma samples compared to normal controls, reinforcing its potential as a diagnostic biomarker in lung cancer [69]. In addition, lower miR-497 levels are associated with poorer OS, supporting its prognostic relevance in NSCLC [70]. In NSCLC, miR-497 has been indicated to target several genes such as yes-associated protein 1 (*YAP1*) [70], cyclin E1 (*CCNE1*) [71] and *Bcl-2* [72]. Notably, *Bcl-2* repression plays a key role in modulating platinum chemoresistance [72].

We also studied the expression of key immune-related genes and found increased expression of *TIGIT*, *FOXP3*, *CXCL10*, and *PD-L1* but not *CD8A* in tumor vs. normal tissues, indicating increased infiltration of immune cells other than cytotoxic CD8^+^ T cells. Interestingly, increased *PD-L1* expression modestly but positively correlated with the expression of miR-26a and miR-30e-3p from our six-miRNA signature. Considering the previously described chemosensitizing role of these miRNAs, it is plausible to hypothesize that, besides apoptosis, these miRNAs may augment the effect of chemotherapy by also modulating the immune microenvironment, in the context of DNA damage response [73]. Of note, miRNAs, including the miR-34 family, have emerged as potential regulators of the PD-1/PD-L1 signaling axis, influencing the sensitivity of NSCLC patients to immune checkpoint blockade [74]. Our results are preliminary, warranting further validation in larger cohorts that will also include patients exposed to adjuvant immunotherapy in addition to chemotherapy.

An important aspect of the present study is the integrated evaluation of the same six-miRNA signature across multiple clinically relevant dimensions. Specifically, we assessed its diagnostic and prognostic significance in tumor tissue, examined the corresponding miRNAs in paired plasma samples, and explored their associations with selected immune-related features of the TME. The combined analysis of tissue and plasma extends our previous findings on platinum sensitivity and strengthens the translational relevance of the signature, particularly given the growing interest in liquid biopsy as a minimally invasive approach for disease monitoring. The detection of these miRNAs in circulation further supports their potential as minimally invasive biomarkers for patient stratification. Particularly, liquid biopsy offers several advantages over repeated tissue sampling, including minimal invasiveness, easier accessibility, and the possibility of serial sampling for longitudinal disease monitoring. However, circulating miRNA analysis remains limited by pre-analytical and methodological variability, low RNA abundance, and potential contributions from non-tumor sources, which currently hinder its standardization and clinical implementation [75]. While our results are promising, the sample size, particularly for plasma, was relatively small, precluding correlation analysis and warranting further validation in larger, independent cohorts. Additionally, functional studies are needed to delineate the precise mechanistic roles of each miRNA in modulating cisplatin response and immune activation within the TME. Longitudinal monitoring of plasma miRNA levels in patients exposed to cisplatin-based chemotherapy may provide valuable further insights into the regulation of each miRNA in responders and non-responders.

## 5. Conclusions

The present study supports the potential diagnostic and prognostic relevance of a six-miRNA signature in resected NSCLC treated with platinum-based chemotherapy. The signature discriminated tumor from matched non-malignant tissue and was associated with RFS, while detection of its components in plasma supports further investigation of their potential as minimally invasive biomarkers. Associations between selected miRNAs and *PD-L1* expression additionally suggest a possible relationship with the tumor immune microenvironment. However, given the exploratory nature and limited size of the study, validation in larger, independent cohorts is essential to further validate its clinical utility and support its integration into precision oncology strategies for NSCLC.

## Figures and Tables

**Table 1:** Univariate and multivariate analysis of tumor tissue miRNA expression for RFS and OS in patients with NSCLC.

	Univariate Analysis			
	RFS		OS	
	HR (95% CI)	*p* Value	HR (95% CI)	*p* Value
miR-26a high vs. low	1.810 (0.903–3.629)	0.094	0.727 (0.202–2.611)	0.625
miR-34a high vs. low	0.719 (0.370–1.399)	0.332	1.405 (0.387–5.106)	0.605
miR-29c high vs. low	1.662 (0.850–3.251)	0.137	1.044 (0.315–3.460)	0.944
miR-497 high vs. low	0.936 (0.481–1.822)	0.845	0.898 (0.271–2.982)	0.861
miR-30e-3p high vs. low	1.166 (0.592–2.296)	0.657	0.453 (0.127–1.620)	0.223
miR-30e-5p high vs. low	1.127 (0.574–2.212)	0.729	2.954 (0.741–11.777)	0.125
	Multivariate Analysis			
	RFS		OS	
	HR (95% CI)	*p* Value	HR (95% CI)	*p* Value
miR-26a high vs. low	2.777 (1.121–6.877)	0.027	0.802 (0.153–4.217)	0.795
miR-34a high vs. low	0.248 (0.089–0.694)	0.008	0.853 (0.155–4.684)	0.854
miR-29c high vs. low	2.890 (1.185–7.049)	0.020	4.464 (0.758–26.307)	0.098
miR-497 high vs. low	0.537 (0.226–1.274)	0.158	0.483 (0.119–1.955)	0.307
miR-30e-3p high vs. low	0.797 (0.308–2.063)	0.640	0.199 (0.028–1.396)	0.104
miR-30e-5p high vs. low	3.080(1.073–8.837)	0.036	10.293 (1.274–83.154)	0.029

## Data Availability

The publicly available datasets analyzed in this study are displayed in Table S1. Patients’ data analysis will be available upon request.

## Supplementary Materials

The following supporting information can be downloaded at https://www.mdpi.com/article/10.3390/cancers18203351/s1. Table S1: Patients’ clinicopathological characteristics; Table S2: Assay ID for each miRNA used in the study. Table S3: Assay ID for each gene used in the study; Figure S1: Kaplan–Meier analysis for RFS based on tissue miR-26a (**A**), miR-34a (**B**), miR-29c (**C**), miR-497 (**D**), miR-30e-3p (**E**) and miR-30e-5p (**F**) expression levels; Figure S2: Kaplan–Meier analysis for OS based on the tissue miR-26a (**A**), miR-34a (**B**), miR-29c (**C**), miR-497 (**D**), miR-30e-3p (**E**) and miR-30e-5p (**F**) expression levels; Figure S3: Kaplan–Meier analysis for RFS based on plasma miR-26a (**A**), miR-34a (**B**), miR-29c (**C**), miR-497 (**D**), miR-30e-3p (**E**) and miR-30e-5p (**F**) expression levels; Figure S4: Kaplan–Meier analysis for OS based on plasma miR-26a (**A**), miR-34a (**B**), miR-29c (**C**), miR-497 (**D**), miR-30e-3p (**E**) and miR-30e-5p (**F**) expression levels.

## Abbreviations

The following abbreviations are used in this manuscript:

ADC	Adenocarcinoma
AUC	Area Under the Curve
CCNE1	Cyclin E1
CDNA	Complementary DNA
CI	Confidence Interval
Ct	Cycle Threshold
CTCs	Circulating Tumor Cells
ctDNA	Circulating Tumor DNA
DDR	DNA Damage Repair
EDTA	Ethylenediaminetetraacetic Acid
ΕΜΤ	Εpithelial-to-Μesenchymal Τransition
FFPE	Formalin-Fixed Paraffin-Embedded Tissue
HR	Hazard Ratio
MiRNAs	MicroRNAs
NSCLC	Non-Small-Cell Lung Cancer
RT-qPCR	Quantitative Real-Time PCR
OS	Overall Survival
RFS	Relapse-Free Survival
ROC	Receiver Operating Curve
SqCC	Squamous Cell Carcinoma
TMB	Tumor Μutational Burden
TME	Tumor Microenvironment
YAP1	Yes-Associated Protein 1
