# Supplementary material for: Evaluation of the Diagnostic and Prognostic Potential, and Correlation with the Tumor Immune Microenvironment of a Six-miRNA Signature Associated with Platinum Sensitivity in Patients with Early-Stage Non-Small-Cell Lung Cancer
Source: Cancers (Basel). 2026 Oct 16;18(20):3351. doi: 10.3390/cancers18203351 (PMC13702743; doi:10.3390/cancers18203351)
Supplement: Supplementary file 1 [file cancers-18-03351-s001.zip › cancers-4605179-supplementary.pdf]

cancers MDPI

Supplementary Materials: Evaluation of the Diagnostic and Prognostic Potential, and Correlation with the Tumor Immune Microenvironment of a Six-miRNA Signature Associated with Platinum Sensitivity in Patients with Early-Stage Non-Small-Cell Lung Cancer

Maria Mortoglou, Chara Papadaki, Aristeidis E. Boukouris, Kleita Michaelidou, Eleftherios Vorrias, Maria Kamaratou, Georgios Koronakis, Eleni Lagoudaki, Anastasios Koutsopoulos, Dimitrios Mavroudis and Sofia Agelaki

Table S1. Patient clinicopathological characteristics.

	Patients	
	**N=76**	**%**
**Characteristic**		
**Gender**		
Male	66	86.8
Female	10	13.2
**Age**		
<70	55	72.4
≥70	21	27.6
**Stage**		
IA	7	9.2
IB	11	14.5
IIA	14	18.4
IIB	19	25
IIIA	22	28.9
IIIB	3	3.9
**Grade**		
1 (Well differentiated)	4	5.3
2 (Moderately differentiated)	17	22.4
3 (Poorly differentiated)	47	61.8
Unknown	8	10.5
**Tumor Size**		
≤3 cm	29	38.2
>3 - ≤7 cm	38	50
>7 cm	9	11.8
**Smoking Status**		
Current smoker	38	50
Former smoker	17	22.4
Non-smoker	5	6.6
Unknown	16	21.1
**Histotype**		
Adenocarcinoma (ADC)	39	51.3
Squamous cell carcinoma (SCC)	32	42.1
Pleomorphic ADC	1	1.3
Large Cell	3	3.9
Unknown	1	1.3
**Lymph Node (LN) status**		

NX	-	-
N0	32	42.1
N1	24	31.6
N2	15	19.7
N3	-	-
N1&N2	5	6.6
**Relapse**		
Yes	39	51.3
No	37	48.8
**Survival**		
Dead	58	76.3
Alive (up to last follow-up)	18	23.7

Table S2. Assay ID for each miRNA used in the study.

Assay name	Assay ID
hsa-miR-26a-5p	000405
hsa-miR-29c-5p	001818
hsa-miR-34a-5p	000426
hsa-miR-30e-5p	002223
hsa-miR-30e-3p	000422
hsa-miR-497-5P	001043
hsa-miR-1228-3p	002919
cel-miR-39	000200
U6 snRNA	001973

Table S3. Assay ID for each gene used in the study.

Assay name	Assay ID
TIGIT	Hs01574692_m1
CD8A	Hs00233520_m1
FOXP3	Hs01085834_m1
CXCL10	Hs00171042_m1
PD-L1 (Cd274)	Hs01125301_m1

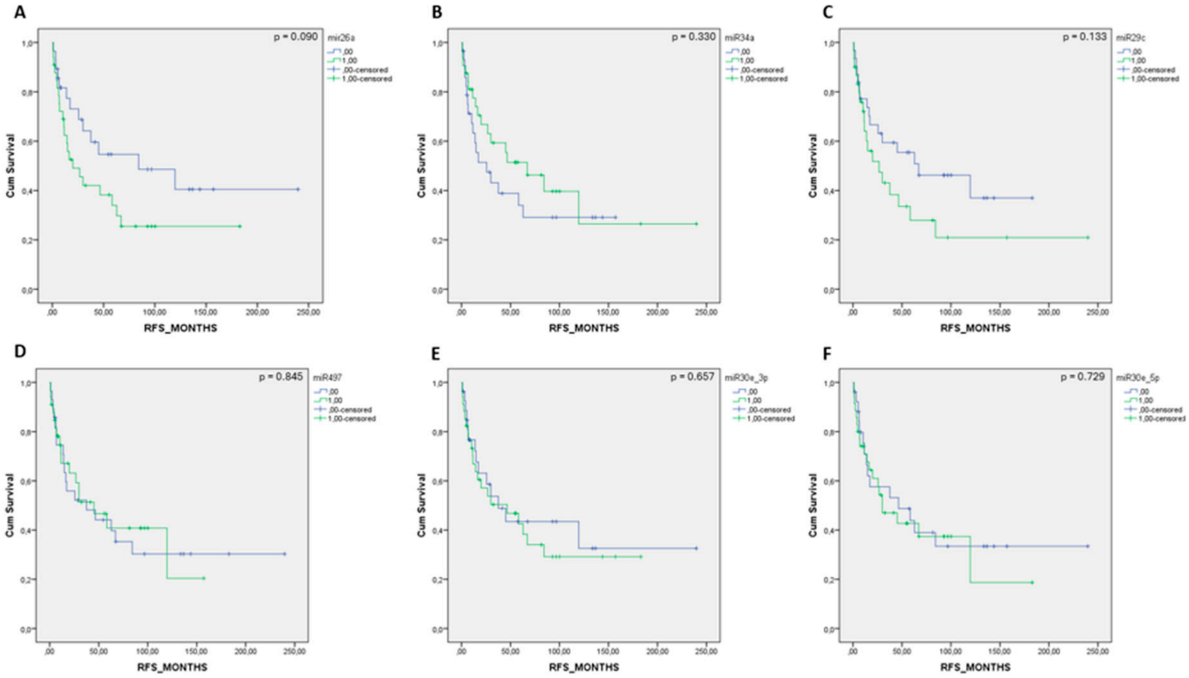

Figure S1. Kaplan–Meier analysis for RFS based on tissue miR-26a (**A**), miR-34a (**B**), miR-29c (**C**), miR-497 (**D**), miR-30e-3p (**E**) and miR-30e-5p (**F**) expression levels. Median expression values classified patients into high (green) and low (purple) expression groups. Curves were compared using the log-rank test. *p*-values are shown.

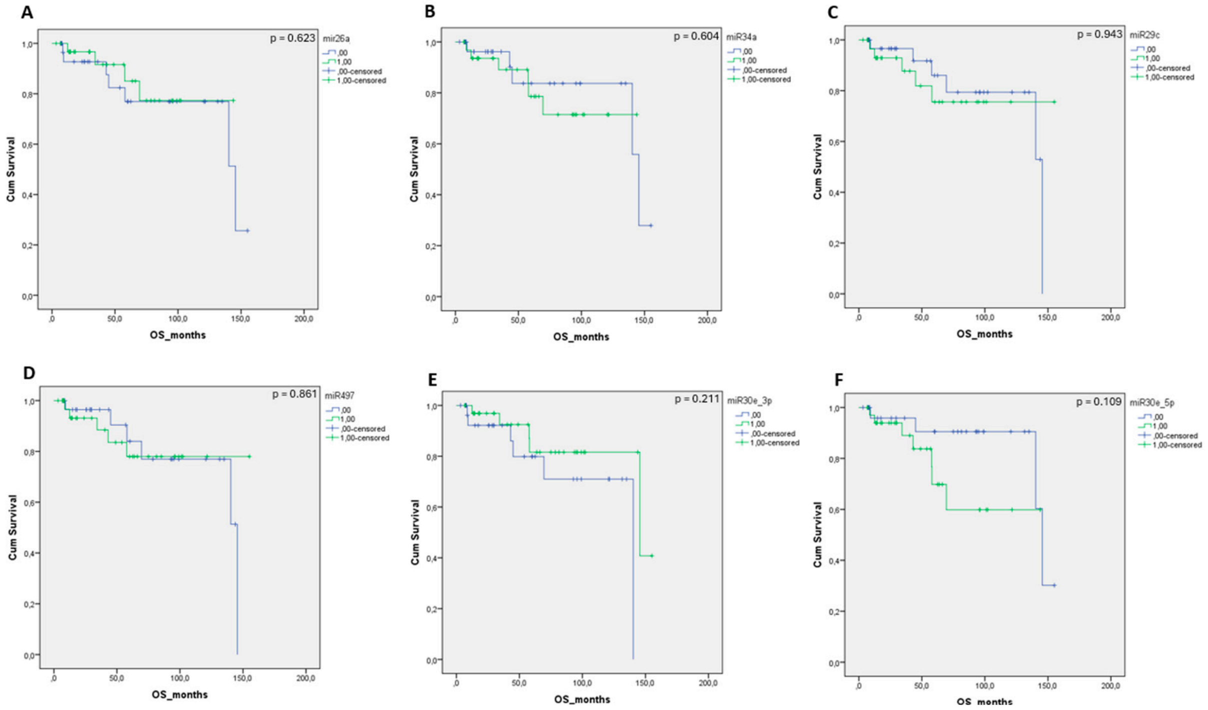

Figure S2. Kaplan–Meier analysis for OS based on the tissue miR-26a (**A**), miR-34a (**B**), miR-29c (**C**), miR-497 (**D**), miR-30e-3p (**E**) and miR-30e-5p (**F**) expression levels. Median expression values classified patients into high (green) and low (purple) expression groups. Curves were compared using the log-rank test. *p*-values are shown.

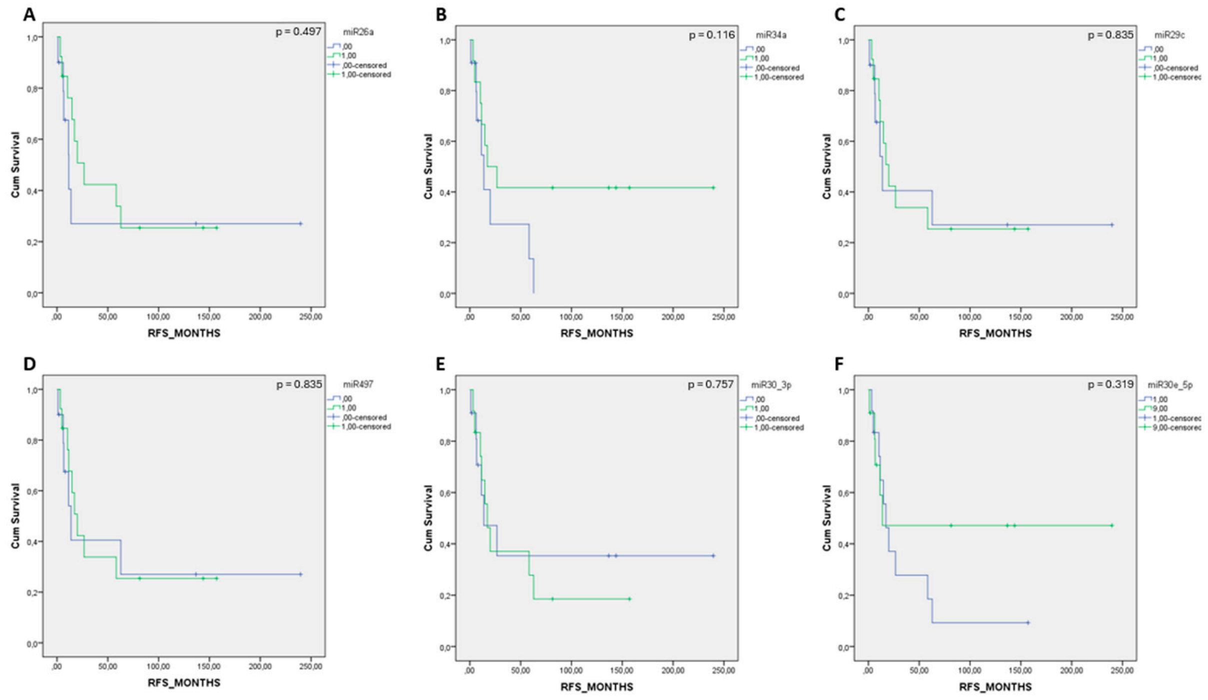

Figure S3. Kaplan–Meier analysis for RFS based on plasma miR-26a (**A**), miR-34a (**B**), miR-29c (**C**), miR-497 (**D**), miR-30e-3p (**E**) and miR-30e-5p (**F**) expression levels. Median expression values classified patients into high (green) and low (purple) expression groups. Curves were compared using the log-rank test. *p*-values are shown.

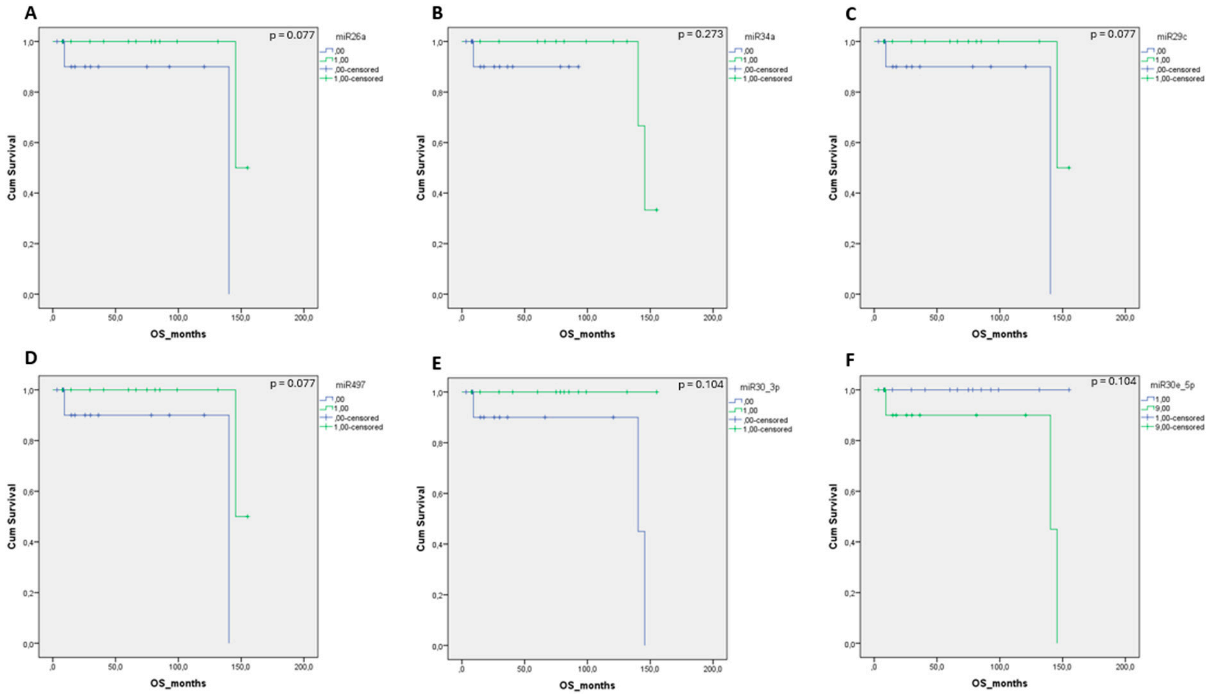

Figure S4. Kaplan–Meier analysis for OS based on plasma miR-26a (**A**), miR-34a (**B**), miR-29c (**C**), miR-497 (**D**), miR-30e-3p (**E**) and miR-30e-5p (**F**) expression levels. Median expression values classified patients into high (green) and low (purple) expression groups. Curves were compared using the log-rank test. *p*-values are shown.
